# Two NAC transcription factors from *Caragana intermedia* altered salt tolerance of the transgenic Arabidopsis

**DOI:** 10.1186/s12870-015-0591-5

**Published:** 2015-08-22

**Authors:** Xiaomin Han, Zongqi Feng, Dandan Xing, Qi Yang, Ruigang Wang, Liwang Qi, Guojing Li

**Affiliations:** College of Life Sciences, Inner Mongolia Agricultural University, Hohhot, 010018 P. R. China; Research Institute of Forestry, Chinese Academy of Forestry, Beijing, 100091 P. R. China

## Abstract

**Background:**

Plants are continuously challenged by different environment stresses, and they vary widely in their adjustability. NAC (NAM, ATAF and CUC) transcription factors are known to be crucial in plants tolerance response to abiotic stresses, such as drought and salinity. ANAC019, ANAC055, and ANAC072, belong to the stress-NAC TFs, confer the Arabidopsis abiotic stress tolerance.

**Results:**

Here we isolated two stress-responsive *NACs*, *CiNAC3* and *CiNAC4*, from *Caragana intermedia*, which were induced by ABA and various abiotic stresses. Localization assays revealed that CiNAC3 and CiNAC4 localized in the nuclei, consistent with their roles as transcription factors. Histochemistry assay using *Pro*_*CiNAC4*_::*GUS* transgenic Arabidopsis showed that the expression of the GUS reporter was observed in many tissues of the transgenic plants, especially in the root vascular system. Overexpression of *CiNAC3* and *CiNAC4* reduced ABA sensitivity during seed germination, and enhanced salt tolerance of the transgenic Arabidopsis.

**Conclusions:**

We characterised *CiNAC3* and *CiNAC4* and found that they were induced by numerous abiotic stresses and ABA. GUS histochemical assay of *CiNAC4* promoter suggested that root, flower and local damaged tissues were the strongest stained tissues. Overexpression assay revealed that CiNAC4 play essential roles not only in promoting lateral roots formation, but also in responding to salinity and ABA treatment of Arabidopsis.

**Electronic supplementary material:**

The online version of this article (doi:10.1186/s12870-015-0591-5) contains supplementary material, which is available to authorized users.

## Background

Many adverse environmental conditions, such as drought, high salinity, extreme temperature, have severe effects on the vegetative growth and development of plants. The decrease of the productivity caused by these abiotic stresses is major challenges for modern agriculture. Plants have evolved various tiers of adaptation mechanisms to unfavorable conditions, including strategies at molecular, cellular, physiological, and biochemical level. The transcriptional activation of a large number of genes upon perception of external stresses include function proteins such as chaperones, the ion transporters, LEA (late embryogenesis abundant) proteins, osmotin, regulatory proteins such as the transcription factors (TFs) [1, 2]. The interactions between transcription factors (TFs) and their corresponding cis-acting elements act as molecular switches for gene expression, directing their temporal and spatial expression [3].

The plant-specific transcription factor NAC family (NAM, ATAF and CUC) shares a conserved NAC domain in the N terminus responsible for DNA binding and diversified C terminal domains for transcription activation [4]. Investigations among several plant species with complete genome sequences have identified 117 NACs in Arabidopsis (*Arabidopsis thaliana*), 151 in rice (*Oryza sativa*) [5], 74 in grape (*Vitis vinifera*) [6], and 152 in soybean (*Glycine max*) [7], which makes the NAC family one of the largest of TFs in plants. Tran *et al*. revealed that *ANAC019*, *ANAC055*, and *ANAC072* responded to abiotic stress and their over-expression conferred improved drought tolerance in Arabidopsis [8]. Thus the roles of NAC family in various abiotic stresses had been noticed as compared to their role in plant development. Puranik *et al*. listed many NAC TFs that influenced plant stress tolerance along with their target genes [9]. Dimerization of TFs can function in modulating the DNA-binding specificity [10]. The NAC domain of approximately 150 amino acids was sufficient to form a homodimer or heterodimer [11]. The consensus sequences that NAC family members were distinguished in most cases with weak base requirements [8, 9, 12–14]. ANAC019, ANAC055, and ANAC072, belong to the stress-NAC TFs [15], and could bind to the promoter of *ERD1* (*EARLY RESPONSIVE TO DEHYDRATION STRESS 1*) by the CATGTG motif and activate its expression [8].

In soybean, Tran *et al*. analyzed 31 full-length *NAC* genes and found that nine were induced by dehydration and exhibited a large diversity in response to high salinity, cold and ABA treatment [16]. Among these 31 *NACs*, *GmNAC2* [17], *GmNAC3*, *GmNAC4* [18], *GmNAC5* [19], *GmNAC6* [20], *GmNAC11* and *GmNAC20* [21] were reported either to be induced by abiotic stresses or to participate in stress toleranc*e. Caragana intermedia* Kuang & H.C. Fu, a leguminous plant, is a native desert shrub with strong drought, salinity, cold resistance, sand-fixing capacity and high forage value. It is extensively distributed in Inner Mongolia, Ningxia Autonomous Regions and Shanxi Province of China [22]. Due to its tolerance to various stresses, *C. intermedia* is an ideal material for studying the mechanism of stress tolerance and can offer effective opportunities for genetic engineering.

In this study, we identified two stress-NAC TFs encoding genes, *CiNAC3* and *CiNAC4*, which were responsive to various abiotic stresses such as dehydtration, drought, salt, cold, heat and wounding. GFP-tagged CiNAC3 and CiNAC4 revealed their nuclear localization. To investigate the *in vivo* functions of CiNAC3 and CiNAC4, we generated transgenic Arabidopsis plants overexpressed *CiNAC3* and *CiNAC4* driven by the *CaMV35S* promoter. The ectopic expression of *CiNAC3* and *CiNAC4* altered ABA sensitivity during seed germination and salt tolerance of the transgenic plants.

## Result

### Identification and cloning of *CiNAC3* and *CiNAC4*

**Fig. 1:**
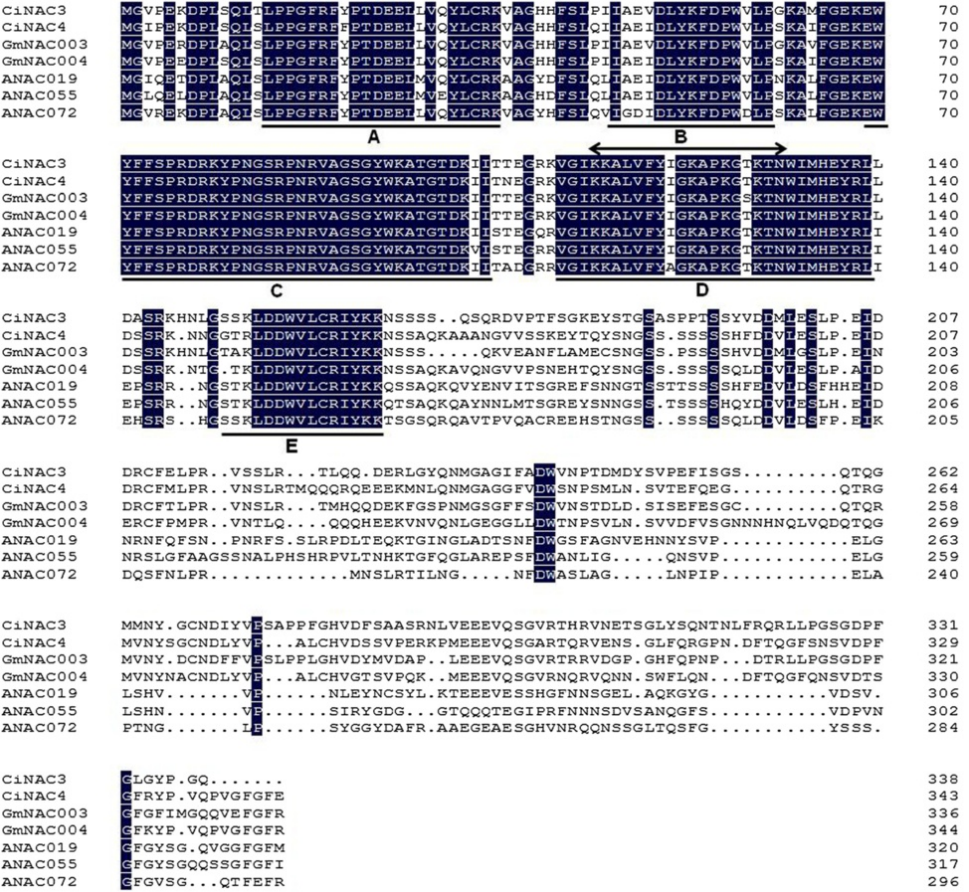
Alignment of the amino acid sequences of some stress-NAC TFs. The putative nuclear localization signal is shown by a double-headed arrow above the sequence. The consensus sub-domains (**a**-**e**) in the NAC binding domain are indicated by underlines. Identical amino acids are indicated by white letters on a black background

**Fig. 2:**
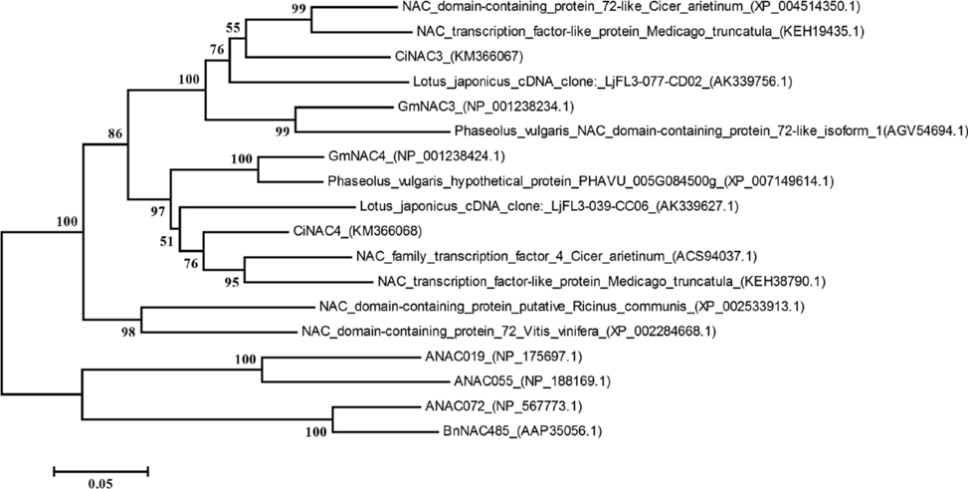
The phylogenetic relationship of the deduced CiNAC3 and CiNAC4 proteins. Phylogenetic analyses were conducted by MEGA5 using the neighbor-joining method. All nodes have 0.80 or greater posterior possibilities. The numbers beside each node represent bootstrap values based on 1,000 replications. The scale bar indicates the relative amount of change along branches

The cDNA and gDNA sequences of *CiNAC3* and *CiNAC4* were isolated by RACE from *C. intermedia* and the intermediate fragments were acquired from the *C. korshinskii* (a very closer species of *C. intermedia*) SSH library under dehydration treatment [23]. Both genes contain three exons and two introns. The putative amino acid sequences were compared with those in the GenBank database by BLAST. CiNAC3 shares identities of 61 % to ANAC072 (RD26) [GenBank: NP_567773.1], and 76 % to GmNAC3 [GenBank: NP_001238234.1], while CiNAC4 has an identities of 59 % to ANAC072, and 80 % to GmNAC4 [GenBank: NP_001238424.1]. A multiple alignment of some stress-NAC TFs [15] by DNAMAN revealed that CiNAC3 and CiNAC4 shared a highly conserved N terminal DNA binding domain, named NAC domain which consists of 5 consensus sub-domains, and a highly variable C-terminal transcriptional regulation domain (Fig. 1). To investigate the divergence of CiNAC3 and CiNAC4 proteins with other NAC proteins during evolution, we analyzed the phylogenetic relationship of CiNAC3 and CiNAC4 with some stress-NAC proteins from *A. thaliana*, *Brassica napus*, *Ricinus communis*, *V. vinifera* and the leguminous plants by MEGA5.1 (Fig. 2). It suggested that CiNAC3 and CiNAC4 belong to the stress-NAC proteins and indicated a regulatory role in stress responses.

### The expression profiles of *CiNAC3* and *CiNAC4* under various stresses

**Fig. 3:**
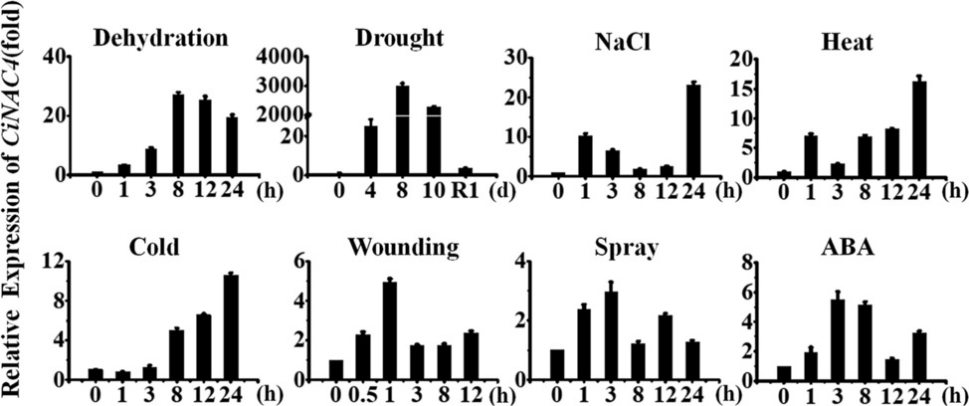
*CiNAC4* were induced by abiotic stress and ABA. One-month-old *C. intermedia* seedlings treated with exogenous ABA (sprayed with 200 μM ABA), cold stress (put into 4 °C incubator), heat stress (put into 42 °C incubator), NaCl (watered with 200 mM NaCl), wounding (2/3 of the total leaves were pierced with tweezers), spray (sprayed with water, used as the control of ABA treatment), dehydration stress (cleaned the soil on the root and put on the filter paper), or drought stress (withholding water) were harvested at the indicated time points. Expression values were calculated using 2^-ΔΔCT^ method and *CiEF1a* as endogenous control. Two independent biological replicates were performed with similar result. Three technical replicates of each biological replicate were analyzed in quantitative real-time PCR analysis

To investigate the response of *CiNAC3* and *CiNAC4* to various abiotic stress, quantitative real time RT-PCR analysis was performed. Both *CiNAC3* and *CiNAC4* were induced by abiotic stresses, such as osmotic stress, salt, wounding, high and low temperature (Fig. 3 and Additional file 1). Particularly, there were hundreds or even thousands fold of changes under drought condition. There exists a distinction between ABA-dependent and ABA-independent pathways for regulating gene expression in response to abiotic stress [1, 9]. We further examined whether *CiNAC3* and *CiNAC4* responded to ABA treatment or not, and found that these two genes were induced within 3 h after application of ABA (Fig. 3 and Additional file 1). These results suggested that CiNAC3 and CiNAC4 might be involved in osmotic stresses and ABA signaling.

### Subcellular localization of CiNAC3 and CiNAC4

**Fig. 4:**
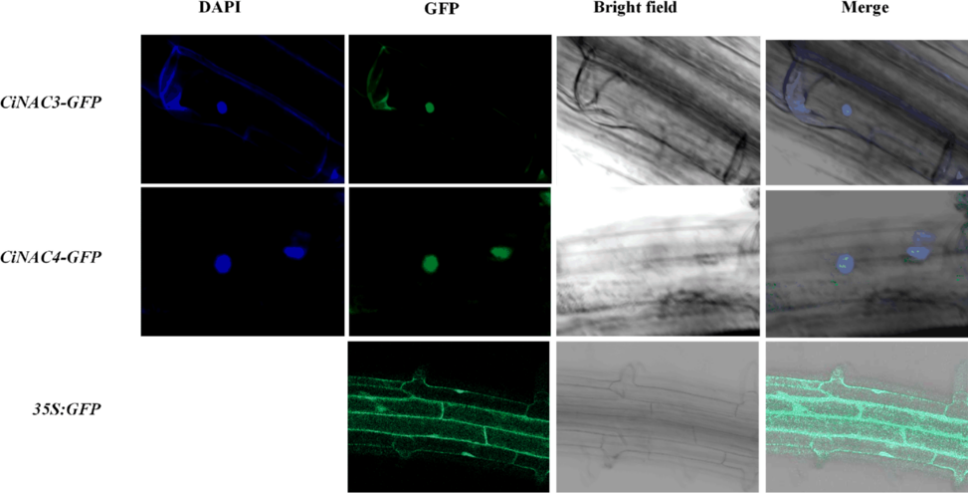
Subcellular localization of CiNAC3 and CiNAC4. Root of the transgenic plants containing the *35S:CiNAC3-GFP* (upper panel), *35S:CiNAC4-GFP* (middle panel), and *35S:GFP* (bottom panel) fusion genes were observed. DAPI was used to visualize the nucleus

To confirm the subcellular localization of CiNAC3 and CiNAC4, the coding region of *CiNAC3* and *CiNAC4* were fused to the C-terminal of *GFP* marker gene, and the fusion genes were driven by the cauliflower mosaic virus (CaMV) 35S promoter. Root tips of the transgenic seedlings were examined for GFP fluorescence. A strong fluorescence signal was predominately observed in the nuclei (Fig. 4). In contrast, the GFP signal distributed throughout the cell in the *35S*::*GFP* transgenic lines. These results were consistent with the role of CiNAC3 and CiNAC4 as TFs.

### Stage and tissue specificity of *CiNAC4* expression

**Fig. 5:**
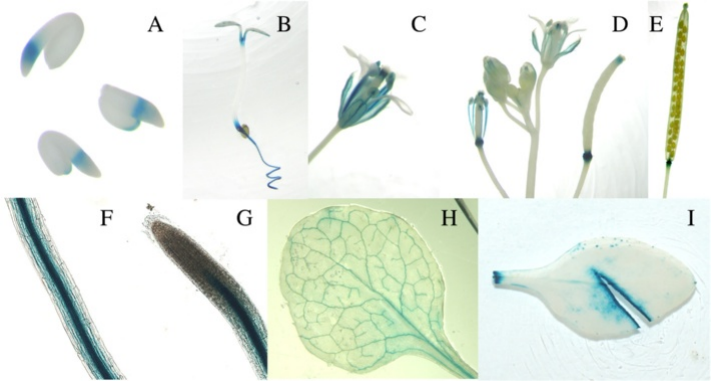
Tissue-specific expression patterns of CiNAC4. **a**, Seeds imbibed for 36-h. **b**, Five-day seedlings. **c**, Flowers. **d**, Inflorescences. **e**, Mature siliques. **f**, Roots. **g**, Root tips. **h** and **i**, Leaves

To investigate the temporal and spatial expression patterns of these two genes in more details, we cloned the 1,143 bp promoter region (the promoter fragment and 5′-untranslated region) of *CiNAC4* using Genome Walking Kit. We failed to clone the promoter of *CiNAC3*. The transgenic lines containing the Pro_*CiNAC4*_::*GUS* construct was used to determine the GUS expression. Histochemical GUS staining was detected, with varying intensity, in many tissues of the transgenic plants (Fig. 5). High GUS expression was limited to the vascular system of root (b, f, g), sepals (c), and in the filament of the stamen (d), but not in root tips (g). The staining was also observed in the cotyledons (b) and both ends of siliques (e) and stigma (d). However, true leaves among the different transgenic lines showed a different pattern. Four out of 19 positive lines showed a strong vein staining (h), while others just showed a slight and smear staining among whole leaves but had a strong GUS activity in the local damaged tissues after wounding treatment (i). The phenotype of wounding induced expression was in accordance with the qPCR result (Fig. 3).

### Overexpression of *CiNAC3* and *CiNAC4* altered ABA sensitivity during seed germination

**Fig. 6:**
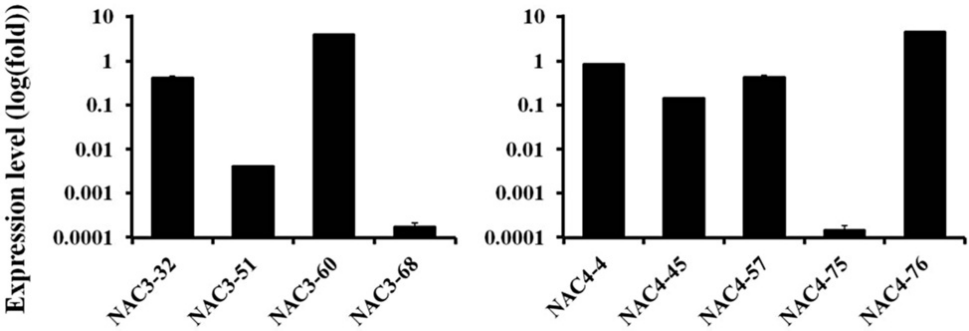
Expression level of *CiNAC3* or *CiNAC4* in their overexpression lines. The T3 transgenic plants growing under normal condition was detected by relative quantitative real-time PCR. There was no amplification in Col-0. Expression values were calculated using 2^-ΔCT ^method and *AtEF1α* as endogenous control. Two independent biological replicates were performed with similar result.Three technical replicates of each biological replicate were analyzed in quantitative real-time PCR analysis

To explore the function of CiNAC3 and CiNAC4 *in planta*, we developed transgenic Arabidopsis constitutively expressing *CiNAC3* or *CiNAC4* genes under control of the 35S promoter. Real-time RT-qPCR was used to detect the transcripts of *CiNAC3* and *CiNAC4* in their overexpression homozygous plants (Fig. 6). Four representative homozygote lines (*NAC3-51*, *NAC3-60*, *NAC4-45*and *NAC4-76)* with different expression levels were used in the following experiments. No notable morphological differences were observed between wild-type and the transgenic plants throughout their life cycle.

**Fig. 7:**
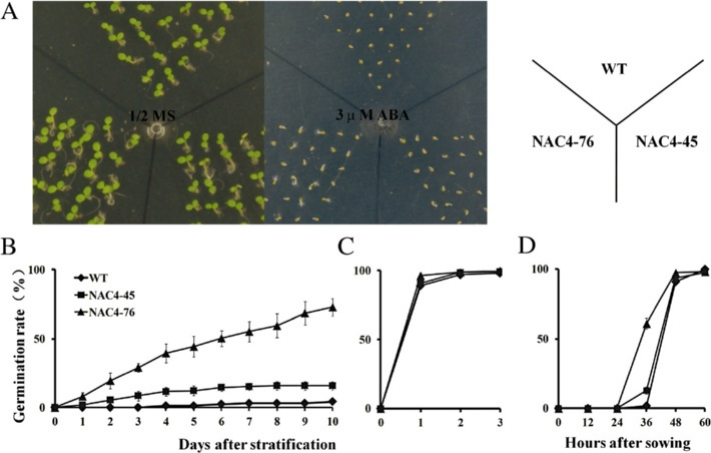
Germination of *CiNAC4* transgenic seeds under ABA treatment. (**a**) The transgenic seeds showed a higher germination rate on 3 μM ABA medium compared with the wild-type. The picture was taken 7 d (3 d for control) after imbibition. The germination rate of wild-type and two overexpression lines on medium with (**b**) or without (**c**) 6 μM ABA. (**d**) Germination of transgenic seeds without stratification. Error bars are standard errors of the means from three replications. Three independent biological replicates have been performed

ABA has been shown to regulate many aspects of plant growth and development, and its role in seed germination has been illustrated [24]. In order to understand whether the ectopic expression of *CiNAC3* or *CiNAC4* altered seed germination phenotype, the germination rates were analyzed under different ABA concentrations (Fig. 7 and Additional file 2). *CiNAC4* transgenic lines could germinate even on the medium with 6ìM ABA (Fig. 7b), while the wild-type was seriously inhibited. The germination rate was comparable in ABA-free medium between the two genotypes (Fig. 7c). Without stratification, the *CiNAC4* overexpression line also exhibited a higher germination rate (Fig. 7d). *CiNAC3* transgenic lines showed a similar phenotype (Additional file 2). These results suggested that CiNAC3 and CiNAC4 counteract the ABA-induced inhibition of seed germination.

### Overexpression of *CiNAC3* and *CiNAC4* increased the expression of *AtMYB2*

**Fig. 8:**
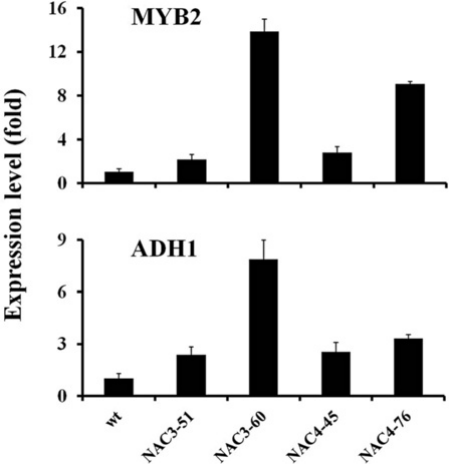
Transcription of *MYB2* and *ADH1* were increased in the transgenic lines. Two-week old seedlings without treatment were harvested. Expression values were calculated using 2^-ΔΔCT ^method and *AtEF1a* as endogenous control. Three independent biological replicates were performed with similar result, and the representative data from one repetition are presented. Three technical replicates of each biological replicate were analyzed in quantitative real-time PCR analysis

Because the *CiNAC3* and *CiNAC4* transgenic plants showed a high germination rate on the medium containing ABA (Fig. 7 and Additional file 2). We detected the downstream genes of ABA biosynthesis and signaling, and found that *AtMYB2*, a positive regulator in ABA signaling [25], was increased in transgenic plants, especially the two lines *NAC3-60* and *NAC4-76* with high expression level (Fig. 8). We searched the microarray data, and the similar result was found that *MYB2* had an expression level of 1.6-fold in the *35S::ANAC055* transgenic plants [8]. *ADH1*, a downstream gene of AtMYB2, was also up-regulated in *CiNAC3* and *CiNAC4* transgenic plants (Fig. 8). This result further confirmed CiNAC3 and CiNAC4 have a function in ABA signaling.

### Overexpression of *CiNAC3* and *CiNAC4* enhanced salt tolerance of the transgenic Arabidopsis

**Fig. 9:**
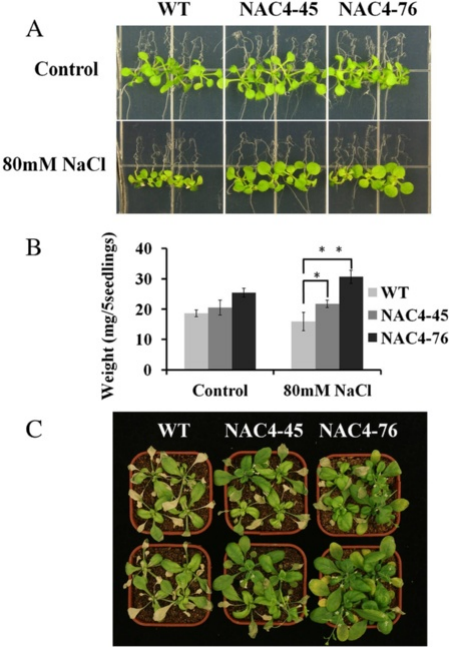
*CiNAC4* overexpression enhanced salt tolerance of the transgenic plants. Five-day old seedlings were transferred to half strength MS medium with or without 80 mM NaCl, and photographed (**a**) and weighed (**b**) after 5 days. (**c**) Four-week old plants were watered with 200 mM NaCl twice, photo was taken after one week

To examine whether the transgenic plants confer tolerance to salt conditions, wild- type and transgenic plants under salt stress were compared. Under normal growth conditions, four transgenic lines showed no obvious abnormal morphological phenotype compared with the wild-type. Five-day old *CiNAC4* transgenic seedlings kept a continuous growth comparing to the wild-type after transferred to half strength MS medium containing 80 mM NaCl (Fig. 9a). The fresh weight was examined and showed a significant difference (Fig. 9b). In addition, *CiNAC4* transgenic plants were allowed to grown in pots for 4 weeks under normal condition, then were watered with 200 mM NaCl and continuous growth was also observed (Fig. 9c). *CiNAC3* transgenic lines also showed great tolerance under watering with 200 mM NaCl (Additional file 3), but the phenotype was not that obvious on half strength MS medium (data not show). These results showed that overexpression of *CiNAC3* and *CiNAC4* in Arabidopsis enhanced tolerance to salt stress.

### Overexpression of *CiNAC4* increased lateral root numbers

**Fig. 10:**
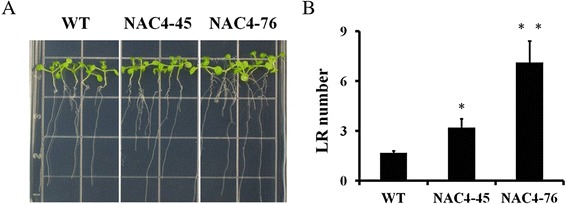
*CiNAC4* overexpression increased lateral root number. The lateral roots of ten-day old seedlings grown on half strength MS media were photographed (**a**) and the number of the lateral roots were counted (**b**). Each data point represents the mean of 15 seedlings. Error bars indicate SD, and asterisks indicate a significant difference (P < 0.05) compared to the control

According to the histochemical assay, *CiNAC4* has a high expression in the vascular system of root. We examined whether CiNAC3 and CiNAC4 could affect the root development. Compared to the wild-type, the *CiNAC4* transgenic plants showed no significant difference in primary root length. However, the lateral root numbers significantly increased in the *CiNAC4* transgenic plants (Fig. 10). This result showed that CiNAC4 promoted lateral root development, and this is consistent with the function of GmNAC4 [18].

## Discussion

To overcome the unfavourable effects of different stresses on crops in agriculture, efforts have been made to enhance the tolerance of crop through both plant breeding and genetic engineering approaches. Ectopic expression of several genes in stress signaling pathways, including NAC TFs, had resulted in different stress tolerance in model plants [9]. The distribution of Caragana species was in the arid and semi-arid area figured their plasticity to the stress conditions. We demonstrated the function of two stress NAC TFs named CiNAC3 and CiNAC4 from *C. intermedia*, and their phylogenetic relationship with other stress NAC TFs from *A. thaliana*, *B. napus, R. communis*, *V. vinifera* and leguminous plants have been compared (Fig. 2). ANAC019, ANAC055, ANAC072 and BnNAC485 were in one clade, and the left NACs were organized in the other clade. In the leguminous clade grouped by *G. max*, *Medicago truncatula*, *Cicer arietinum, Phaseolus vulgaris*, *Lotus japonicas* and *C. intermedia*, there is a distinction between the NAC3 and the NAC4, indicating they might have a different role in certain process. The three ANAC TFs play a key role in the stress-specific gene regulatory network [26]. Then a presumption is that NAC3 and NAC4 may play a comparative role in leguminous plants as the three ANAC TFs in Arabidopsis. It should be noticed that CiNAC3 and CiNAC4 had the closest relationship with the NACs from *C. arietinum* and *M. truncatula*, respectively, and these two species had been known to be tolerant to drought. But further evidence is required.

*CiNAC3* and *CiNAC4* can be induced under abiotic stresses including wounding (Fig. 3). The stress-NAC TFs *ANAC019*, *ANAC055* and *ANAC072* were induced by dehydration and salt, this is in accordance with their regulatory role in abiotic stresses [8, 26, 27]. Here, we also found *CiNAC3* and *CiNAC4* had an accumulation and peaked at 1 h after wound treatment (Fig. 3 and Additional file 1). GUS staining of transgenic plants that harbored a Pro_*CiNAC4*_::*GUS* construct showed an strong activity in the local damaged tissues (Fig. 5). GUS expression pattern of *ATAF1* and *ATAF2*, which belongs to the ATAF subgroup and is a relative closer subgroup with stress-NAC TFs [15], also showed an strong GUS activity in the local damaged tissues [28, 29]. Other ATAFs, such as *OsNAC6* [30] and *StNAC* [31] were also wounding-induced. It has been found that the *ATAFs* participated in both abiotic and biotic stresses [9, 32]. *ATAF1* and its homolog *HvNAC6* in barley positively regulated penetration resistance towards the biotrophic fungus *Blumeria graminis* f.sp. *hordei* (Bgh) [33, 34]. We also checked the expression of defense genes *PR1* and *PDF1.2* in the transgenic Arabidopsis, and found that *CiNAC3* and *CiNAC4* down-regulated *PDF1.2* and slightly up-regulated *PR1* (Additional file 4). This suggests that CiNAC3 and CiNAC4 may have a role in plant defense responses. We did not have the opportunity to check the disease tolerance phenotype yet.

*CiNAC3* and *CiNAC4* could be induced under ABA treatment (Fig. 3 and Additional file 1). The expression of *CiNAC3* and *CiNAC4* under JA, SA, IAA and NAA treatments was comparable with that in the control (Additional file 5). The *CiNAC3* and *CiNAC4* ectopic expression lines also exhibited a high germination rate on the ABA medium (Fig. 7 and Additional file 2). ABA plays an important role in many plant processes such as formation and dormancy of seeds, inhibition of germination, stress response and stomata regulation [24]. A previous study had shown that ANAC072 functioned as a transcriptional activator and played a positive role in ABA sensitivity of seedlings and ABA inducible gene expression under abiotic stress [27]. *ANAC019* also showed a positive role in ABA signaling of seed germination and early seedling development [35]. *BnNAC485*, the homologous gene of *ANAC072*, conferred ABA hypersensitivity in transgenic Arabidopsis [36]. Interestingly, GmNAC3 and GmNAC4 play an inconsistent role in seed germination [18]. Our results showed that both CiNAC3 and CiNAC4 could counteract ABA inhibition of seed germination with a dose-dependent manner (Fig. 7 and Additional file 2).

Previous study had shown that AtMYB2 acted as transcriptional activator in ABA signaling [25]. High expression level of *AtMYB2* was found in the *CiNAC3* and *CiNAC4* transgenic plants, especially the high expression lines *NAC3-60* and *NAC4-76* (Fig. 8), showed a dose-dependent pattern. Abe *et al.* found that overexpression of *AtMYB2* in Arabidopsis resulted in ABA sensitive phenotype during seed germination [25]. One explanation is that the high expression level of *AtMYB2* is not the reason of the high germination rate of *CiNAC3* and *CiNAC4* transgenic plants under ABA treatment. We also assayed the expression of other representative genes in ABA synthesis, catabolism and signaling pathways, such as *ABA1*, *ABA2*, *AAO3*, *ABI1*, *ABI3*, *ABI5*, *ABF1*, *ABF2*, *ABF3*, *ABF4*, and *RGL2*, a DELLA that negatively regulated the seed germination, most of the genes checked showed an increase within one fold in the transgenic plants compare to wild type (Additional file 6A). It has been reported that GmNAC3 and GmNAC4 regulated ABA signaling genes [18]. This suggests that CiNAC3 and CiNAC4 may also function in a dose-dependent manner and play a role in ABA signaling of seed germination.

*CiNAC3* and *CiNAC4* can be induced by hundreds or even thousands fold under drought treatment, while only over ten-fold elevation under NaCl treatment (Fig. 3 and Additional file 1). Interestingly, *CiNAC3* and *CiNAC4* transgenic plants showed tolerance to NaCl but not to drought (data not shown), this is different from the drought tolerance phenotype of the *ANAC019*, *ANAC055*, and *ANAC072* overexpression lines [8]. One reason might be the signaling network mediated by NACs in Arabidopsis was distinct from that in *C. intermedia*, although many researchers used Arabidopsis as the model organism to validate the function of their interested genes from various plant species. Currently, we did have trouble in transformation with *C. intermedia*. And *M. truncatula* was also a ideal plants that have close relationship with *C. intermedia*. We are now trying to set up the successful transformantion system of *M. truncatula*.

Osmotic stress such as dehydration, drought, salt, and cold has a critical effect on plant growth and development. For survival, plant evolved some adaptive mechanism that included accumulation of osmolytes, maintenance of the ion homeostasis and detoxification [1, 37]. Salt stress causes more toxic ions entering the plants. We also detected some ion channels encoding genes including *HKT*, *NHX*, *SOS1*, and *SOS2*, which have been proved to be involved in balancing the ion homeostasis, and no more than 2.5 fold changes were found (Additional file 6B). Some reproducible stress responsive marker genes include *COR15A*, *COR47*, *RD22*, *ERD1*, and *ERA1*, also had no remarkable changes in the transgenic plants (Additional file 6C).

The promotion of numbers and length of lateral roots in response to water deficit, is considered as an avoidance mechanism of soybean plants to water stress [38]. Several NAC TFs have been found to respond to environmental stresses and promote lateral root development [21, 39, 40]. We found that the lateral root number of the *CiNAC4* transgenic lines were significantly enhanced compared to wild-type (Fig. 10). This result was in accordance with Quach et al. [18], that GmNAC4 significantly promoted LR number under non-stress and mild water deficit conditions. CiNAC3 and GmNAC3 did not promote LR number [18]. This suggests that CiNAC3 and CiNAC4 play a partially redundant role in plant stress responsive signaling, and their roles in lateral root development process are different.

## Conclusions

We identified two stress-NAC TFs, CiNAC3 and CiNAC4, and found that their transcripts accumulated under various abiotic stresses and ABA treatment. Both TFs were localized in nuclei. The GUS staining experiments indicated that the *CiNAC4* promoter have a high activity in root, flower and local damaged tissues of Arabidopsis. CiNAC3 and CiNAC4 altered the ABA signaling and NaCl tolerance of the transgenic Arabidopsis, while only *CiNAC4* overexpression increased the lateral root number.

## Methods

### Growth conditions and treatments

The Arabidopsis wild-type (Columbia-0) and the transgenic lines were grown on half strength MS medium or a 1:1 mixture of peat soil and vermiculite under long-day conditions (16-h-light/8-h-dark cycle) at 22 °C. To determine the germination rate, seeds were surface sterilized and sown on half strength MS containing 0.65 % agar powder and 3 % sucrose with different concentrations of NaCl or ABA. Germination rates were scored based on radicle protrusion after three days of stratification. Seeds of *C. intermedia* were collected from Hohhot, Inner Mongolia, China. One-month-old seedlings which were sown in pots containing a soil mixture were used to detect the transcript level of *CiNAC3* and *CiNAC4* under various treatments.

For NaCl treatment, the seedlings growing under normal conditions were watered with 200 mM NaCl. For cold and heat treatments, the seedlings were transferred to the 4 °C or 42 °C incubator. For ABA treatment, the seedlings were sprayed with 200 μM ABA plus 0.05 % Tween. For the “spray” treatment which was used as the control of ABA treatment, the seedlings were sprayed with only water containing 0.05 % Tween. For wounding treatment, 2/3 of the total leaves of each seedling was pierced with tweezers. For dehydration treatment, soil was removed and the seedlings were cleaned with tap water, then placed on the filter paper at room temperature. For drought treatment, the seedlings were subjected to drought conditions by withholding water for ten days and then re-watered. Each sample contains three seedlings and the shoots (including stems and leaves) were taken as samples.

### RNA extraction and real-time RT-qPCR analysis

Total RNA was isolated according to manufactures’ instructions (Invitrogen) using Trizol reagent. After DNase I (Ambion Cat# AM2224) treatment, 500 ng (for Arabidopsis) or 1ìg (for Caragana) of RNA was used for reverse transcription (TaKaRa, Dalian, China Cat# D2640A). The cDNA was diluted 40 times (for Arabidopsis) or 16 times (for Caragana), and 5 μL was used as a template in a 20-μL PCR reaction. Real-time PCR analysis was performed using SYBR Green Perfect mix (TaKaRa, Cat# DRR041A) on a LightCycler 480 system (Roche), with the program of 40 cycles under the following conditions: 95 °C for 5 s, 60 °C for 30 s, and 72 °C for 15 s. *AtEF1α* and *CiEF1α* [GenBank: KC679842] was used to normalize the Arabidopsis or Caragana samples respectively [41]. The primers used in this study are listed in Additional file 7.

### Identification of *CiNAC3* and *CiNAC4*

The cDNA intermediate fragments of *CiNAC3* and *CiNAC4* were acquired from a Suppression Subtractive Hybridization (SSH) library of *C. korshinskii* (a very closer species of *C. intermedia*) under dehydration stress which was constructed by Yang *et al*. [23]. Full length cDNA was obtained by Rapid Amplification of cDNA Ends (RACE) using the mRNA extracted from *C. intermedia* as template (TaKaRa, Dalian, China Cat#6107 and Cat#6106). The promoter of *CiNAC4* was cloned using Genome Walking Kit (TaKaRa, Dalian, China Cat#6108). The primers used in this study are listed in Additional file 7: Table S1. PCR for the cloning of *CiNAC3* and *CiNAC4* was performed with the following cycling profile: 98 °C for 2 min; 35 cycles at 98 °C for 10 s, 57 °C for 15 s, and 72 °C for 1.5 min; and a final extension for 10 min at 72 °C. PCR for the cloning of the *CiNAC4* promoter was : 98 °C for 2 min; 35 cycles at 98 °C for 10 s, 59 °C for 15 s, and 72 °C for 1.5 min; and a final extension for 10 min at 72 °C.

Protein sequences were aligned using the DANMAN. Phylogenetic tree was conducted by MEGA5 using the Neighbor-Joining (NJ) method and bootstrap analysis of 1000 replications.

### Construction of *CiNAC3* and *CiNAC4* transgenic plants

The open reading frames (without the termination codon) of *CiNAC3* and *CiNAC4* were cloned into pENTR/D-TOPO (Invitrogen, Cat.#K2420-20). The primer were designed following the direction of pENTR™ Directional TOPO® Cloning Kits, and “CACC” was added to the 5′ end of the sense primer to serve as a recombination site for introducing the PCR product into the entry plasmid, pENTR/D-TOPO. After sequencing and validation of the entry plasmid, the LR recombination reaction was performed between the entry plasmid and the gateway destination vector (Invitrogen Cat.#11791-020). The vector pMDC32 was used for overexpression construct, while pMDC43 was used for C-terminal GFP fused construct to detect the subcellular localization.

The promoter of *CiNAC4* was amplified with high fidelity enzyme and cloned into p*EASY*-Blunt Simple (TransGen, Cat.#CB111-02). After validation, the promoter was fused with the *GUS* reporter gene of pCAMBIA1305.2 with the *CaMV35S* promoter removed using *Hin*dIII and *Nco*I restriction sites.

The binary plasmids were transformed into *Agrobacterium tumefaciens* strain GV3101 by electroporation, which was used for floral dip transformation of Arabidopsis. Transformants were selected on half strength MS medium containing the appropriate antibiotics.

### Histochemical GUS Staining

The T_2_ seedlings of Pro_Ci*NAC4*_::*GUS* transgenic plants were used to determine the GUS activity. The GUS staining protocol was performed as previously described [42]. The images were taken under the dissecting microscope (SMZ800, Nikon).

### Subcellular localization

The T_2_ seeds of *35S*::*GFP-CiNAC3* or *35S*::*GFP-CiNAC4* transgenic lines were grown on half strength MS medium containing 13 mg/L hygromycin B. The root tips of 10-day-old positive seedlings were observed under laser scanning confocal microscope (Zeiss LSM 510).

## Additional files

Additional file 1:
***CiNAC3*** **was induced by abiotic stresses and ABA.** One-month-old *C. intermedia* seedlings treated with exogenous ABA (sprayed with 200 μM ABA), cold stress (put into 4 °C incubator), heat stress (put into 42 °C incubator), NaCl (watered with 200 mM NaCl), wounding (2/3 of the total leaves were pierced with tweezers), spray (sprayed with water, used as the control of ABA treatment), dehydration stress (cleaned the soil on the root and put on the filter paper), or drought stress (withholding water) were harvested at the indicated time points. Expression values were calculated using 2-ΔΔCT method and *CiEF1a* as endogenous control. Two independent biological replicates were performed with similar result. Three technical replicates of each biological replicate were analyzed in quantitative real-time PCR analysis. (TIFF 521 kb) Additional file 2:
**Germination of the** ***CiNAC3*** **transgenic seeds under ABA treatment.** (A) The transgenic lines showed a higher germination rate on 3 μM ABA medium compared with wild-type. The picture was taken 7 d (3 d for control) after imbibition. The germination rate of wild-type and two overexpression lines on medium with (B) or without (C) 6 μM ABA. (D) Germination of transgenic seeds without stratification. Error bars are standard errors of the means from three replications. Three independent biological replicates have been performed. (TIFF 1072 kb) Additional file 3:
***CiNAC3*** **overexpression altered salt tolerance of the transgenic plants.** Four-week old wild-type and *CiNAC3* overexpression plants were watered with 200 mM NaCl twice, photo was taken after one week. (TIFF 1071 kb) Additional file 4:
**Expression of some defence genes in the transgenic plants.** *PDF1.2* was down-regulated and *PR1* was slightly up-regulated in transgenic Arabidopsis. Expression values were calculated using 2^-ΔΔCT^ method and *AtEF1a* as endogenous control. Two independent biological replicates were performed with similar result. Three technical replicates of each biological replicate were analyzed in quantitative real-time PCR analysis. (TIFF 37 kb) Additional file 5:
**Expression of** ***CiNAC3*** **and** ***CiNAC4*** **under various hormones treatment.** One-month-old *C. intermedia* seedlings were sprayed with water, MeJA (100 μM), SA (1 mM), IAA (10 μM), NAA (10 μM). Samples were harvested at the indicated times. Expression values were calculated using 2^-ΔΔCT^ method and *CiEF1a* as endogenous control. Two independent biological replicates were performed with similar result. Three technical replicates of each biological replicate were analyzed in quantitative real-time PCR analysis. (TIFF 89 kb) Additional file 6:
**Stress responsive marker genes profile by quantitative real-time PCR analysis in the transgenic plants.** ABA signaling genes (A), salt signaling genes and some stress responsive genes (B), (C) were detected. Two-week old seedlings with or without treatment were harvested. Expression values were calculated using 2^-ΔΔCT^ method and *AtEF1a* as endogenous control. Two independent biological replicates were performed with similar result. Three technical replicates of each biological replicate were analyzed in quantitative real-time PCR analysis. (TIFF 212 kb) Additional file 7:
**Primers used in this study.** (XLSX 15 kb)

## Abbreviations

NAC: NAM/ATAF/CUC
ABA: Abscissic acid
JA: Jasmonic acid
SA: Salicylic acid
IAA: Indoleacetic acid
NAA: Naphthaleneacetic acid
LEA: Late embryogenesis abundant
TF: Transcription factor
*ERD1*: *Early responsive to dehydration stress 1*
Ci: *Caragana intermedia*
Gm: *Glycine max*
*Hv*: *Hordeum vulgare*
Bn: *Brassica napus*
*CaMV35S*: cauliflower mosaic virus 35S
SSH: Suppression Subtractive Hybridization
*ABI*: *ABA insensitive*
*ABF*: *ABRE binding factor*
*AAO*: *Aldehyde oxidase*
*RGL*: *RGA (repressor of ga)-like*
*COR*: *Cold-regulated*
*RD*: *Response to dehydration*
*HKT1*: *High-affinity K*^*+*^*transporter 1*
*NHX*: *Na*^*+*^*/H*^*+*^*exchanger*
*SOS*: *Salt overly sensitive*
RACE: Rapid Amplification of cDNA Ends
LR: Lateral root
