# Supplementary figures and images for: Two NAC transcription factors from Caragana intermedia altered salt tolerance of the transgenic Arabidopsis
Source: BMC Plant Biol. 2015 Aug 22;15:208. doi: 10.1186/s12870-015-0591-5 (PMC4546137; doi:10.1186/s12870-015-0591-5)

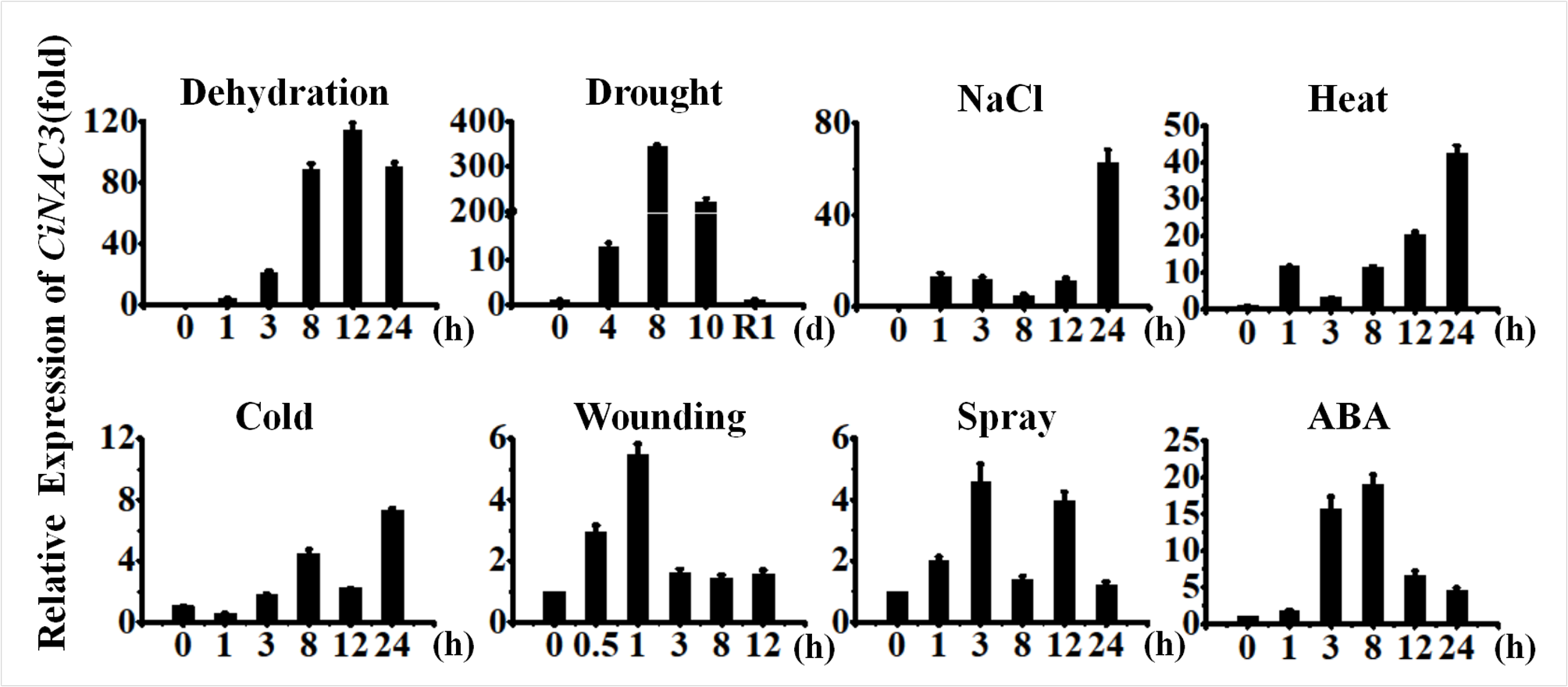

Supplement: Additional file 1: — CiNAC3 was induced by abiotic stresses and ABA. One-month-old C. intermedia seedlings treated with exogenous ABA (sprayed with 200 μM ABA), cold stress (put into 4 °C incubator), heat stress (put into 42 °C incubator), NaCl (watered with 200 mM NaCl), wounding (2/3 of the total leaves were pierced with tweezers), spray (sprayed with water, used as the control of ABA treatment), dehydration stress (cleaned the soil on the root and put on the filter paper), or drought stress (withholding water) were harvested at the indicated time points. Expression values were calculated using 2-ΔΔCT method and CiEF1a as endogenous control. Two independent biological replicates were performed with similar result. Three technical replicates of each biological replicate were analyzed in quantitative real-time PCR analysis. (TIFF 521 kb) [file 12870_2015_591_MOESM1_ESM.tif]

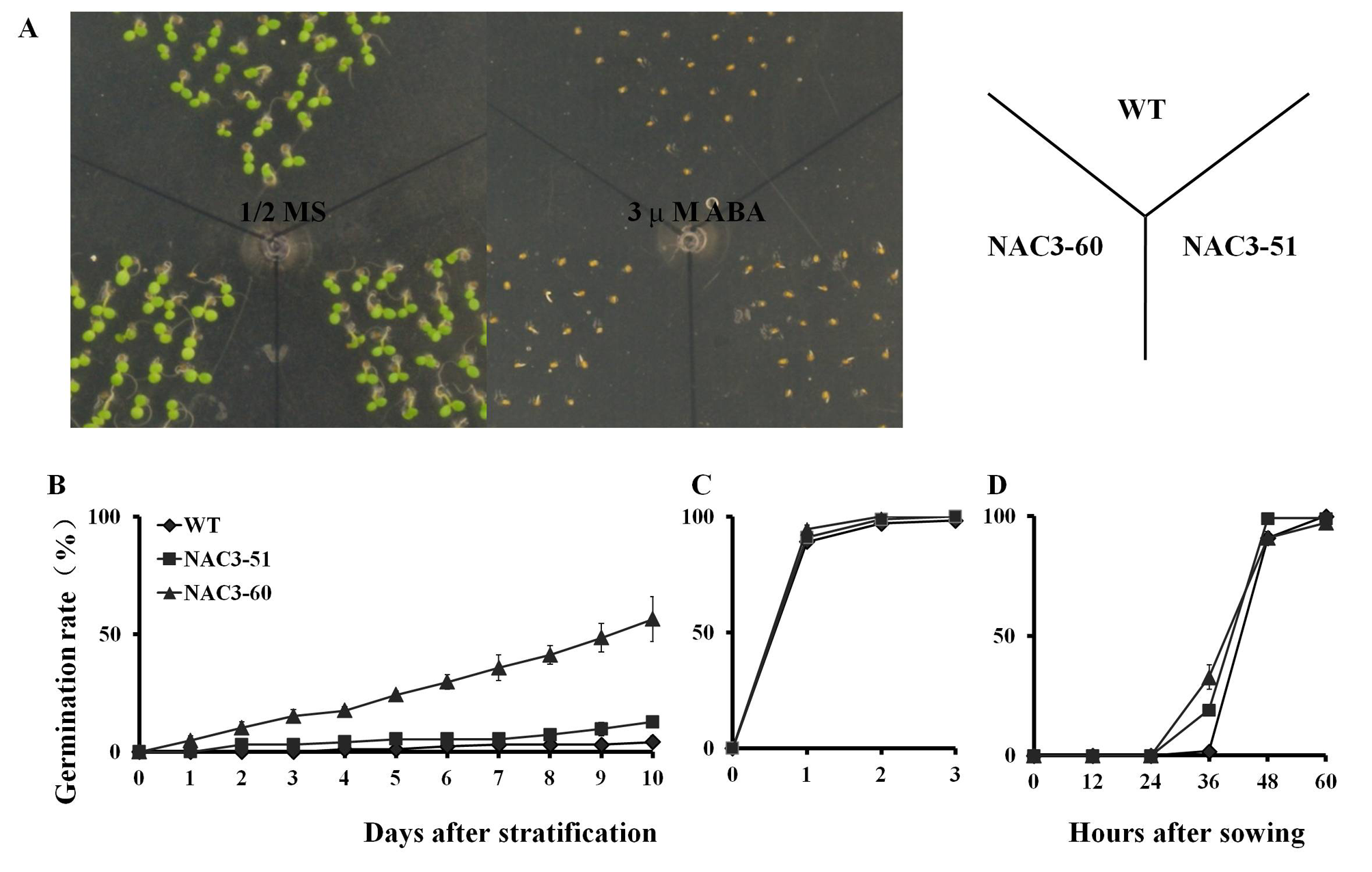

Supplement: Additional file 2: — Germination of the CiNAC3 transgenic seeds under ABA treatment. (A) The transgenic lines showed a higher germination rate on 3 μM ABA medium compared with wild-type. The picture was taken 7 d (3 d for control) after imbibition. The germination rate of wild-type and two overexpression lines on medium with (B) or without (C) 6 μM ABA. (D) Germination of transgenic seeds without stratification. Error bars are standard errors of the means from three replications. Three independent biological replicates have been performed. (TIFF 1072 kb) [file 12870_2015_591_MOESM2_ESM.tif]

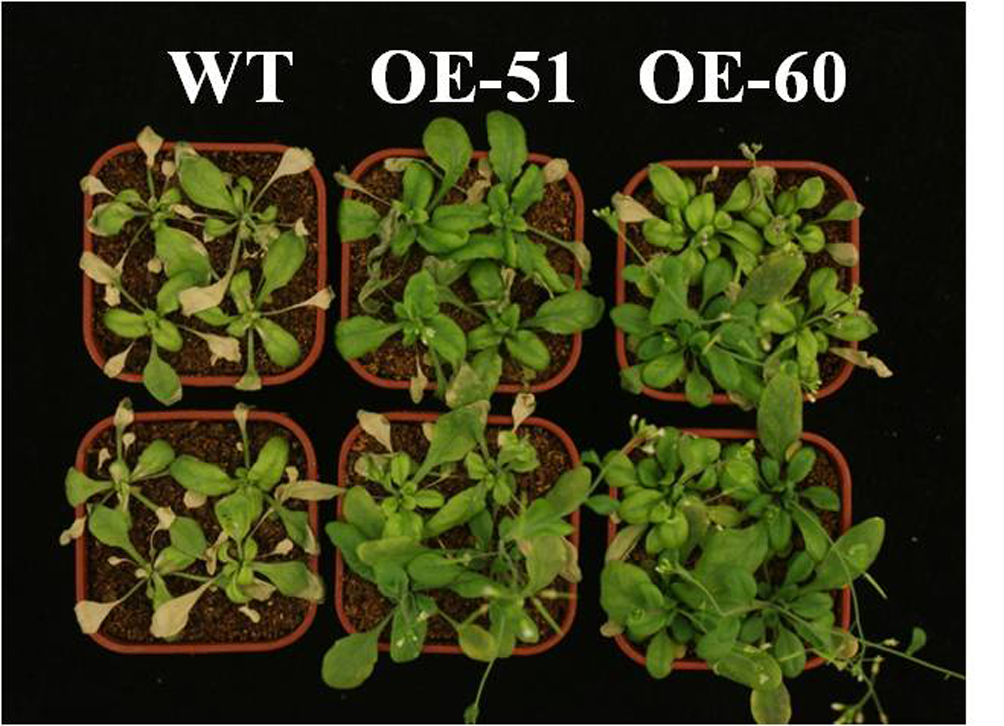

Supplement: Additional file 3: — CiNAC3 overexpression altered salt tolerance of the transgenic plants. Four-week old wild-type and CiNAC3 overexpression plants were watered with 200 mM NaCl twice, photo was taken after one week. (TIFF 1071 kb) [file 12870_2015_591_MOESM3_ESM.tif]

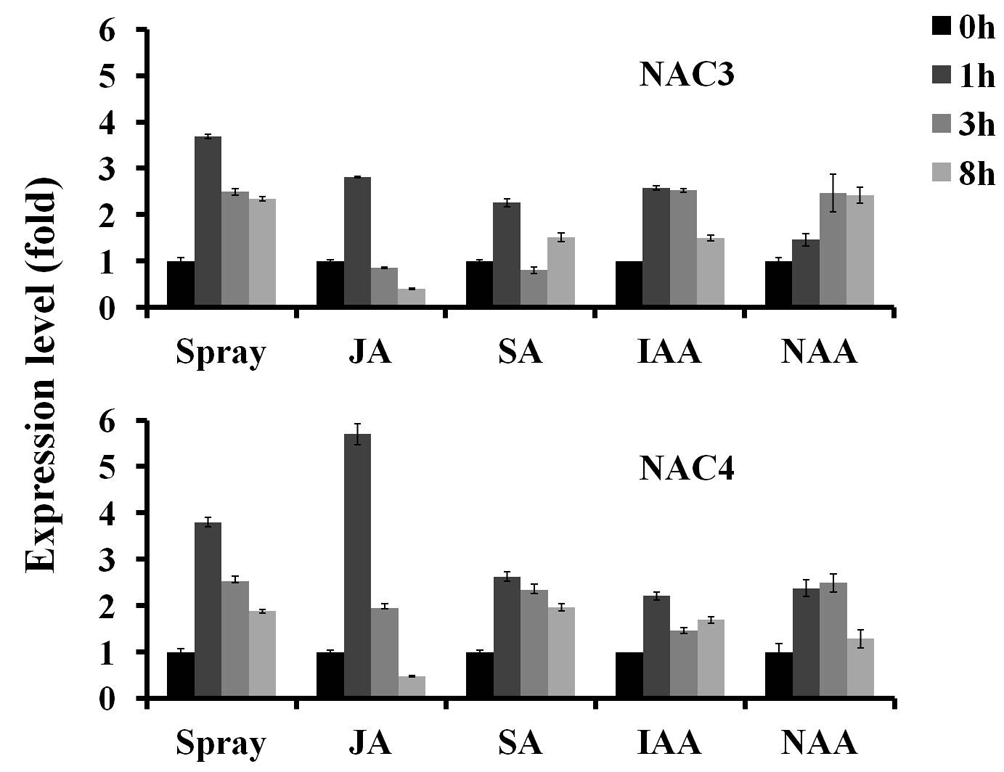

Supplement: Additional file 5: — Expression of CiNAC3 and CiNAC4 under various hormones treatment. One-month-old C. intermedia seedlings were sprayed with water, MeJA (100 μM), SA (1 mM), IAA (10 μM), NAA (10 μM). Samples were harvested at the indicated times. Expression values were calculated using 2-ΔΔCT method and CiEF1a as endogenous control. Two independent biological replicates were performed with similar result. Three technical replicates of each biological replicate were analyzed in quantitative real-time PCR analysis. (TIFF 89 kb) [file 12870_2015_591_MOESM5_ESM.tif]
